# Supplementary material for: Complex‐centric proteome profiling by SEC‐SWATH‐MS
Source: Mol Syst Biol. 2019 Jan 14;15(1):e8438. doi: 10.15252/msb.20188438 (PMC6346213; doi:10.15252/msb.20188438)
Supplement: Supplementary file 7 — Dataset EV6 [file MSB-15-e8438-s007.zip › feature_plots_bioplex/Q9NRD1.pdf]

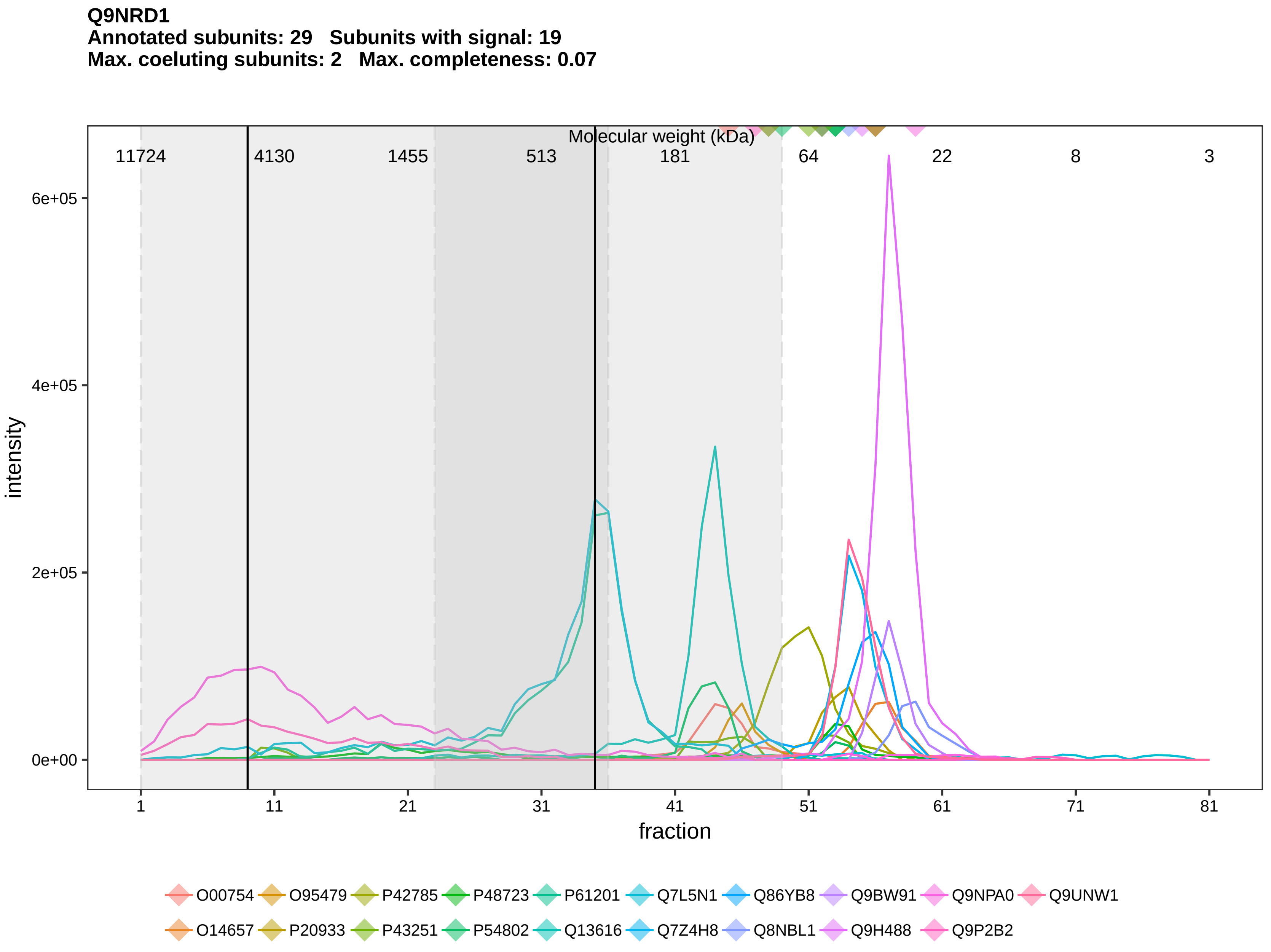
Q9NRD1
Annotated subunits: 29   Subunits with signal: 19
Max. coeluting subunits: 2   Max. completeness: 0.07
Molecular weight (kDa)
11724
4130
1455
513
181
64
22
8
3
intensity
6e+05
4e+05
2e+05
0e+00
fraction
1
11
21
31
41
51
61
71
81
O00754
O95479
P42785
P48723
P61201
Q7L5N1
Q86YB8
Q9BW91
Q9NPA0
Q9UNW1
O14657
P20933
P43251
P54802
Q13616
Q7Z4H8
Q8NBL1
Q9H488
Q9P2B2
